# A web-based system for tissue microarray data management

**DOI:** 10.1186/1746-1596-1-36

**Published:** 2006-10-11

**Authors:** Vincenzo Della Mea, Irene Bin, Maura Pandolfi, Carla Di Loreto

**Affiliations:** 1 Medical Informatics and telemedicine Lab, Dept. of Mathematics and Computer Science, University of Udine, Italy; 2 Section of Pathology, Dept. of Medical Morphological Research, University of Udine, Italy

## Abstract

**Background:**

Tissue Microarray is a novel technique for analysing large amounts of immunohistochemically stained specimens. However, those large amounts make it difficult to design, prepare and analyze a tissue microarray, so that software support is almost inevitable.

**Methods:**

We designed a tissue microarray data management system starting from specifications obtained by pathologists, and arranged for a preliminary validation in thyroid pathology.

**Results:**

A web-based system has been developed, basing on open-source software and principles, that was well accepted by pathologists and allowed to carry out a study on 52 thyroid pathology cases.

**Conclusion:**

Though limited in functionalities, the developed system is effective and can be downloaded at the address .

## Background

Tissue Microarray (TMA) is a novel technique described firstly by Kononen in 1998 [1] that, by collecting up to hundreds of samples on the same specimen, allows to obtain three main advantages:

- reuse of a scarce resource (the tissue block);

- uniformity in staining;

- decreased antibody quantity used per sample.

Although the samples are small (up to some square millimeters), it has been demonstrated in multiple works that they are sufficiently representative of the whole tumour [2-4].

However, the technical preparation of a TMA involves the design of the recipient block, which should host all its samples in recognisable positions. Furthermore, after having prepared and stained a number of glass slides from the block, each sample should be analysed and reported in correspondence with its original patient data. This gives difficulties, due to the large amount of data to be managed in each phase, from TMA design to analysis and final report of results.

At present, just three papers describe software for supporting TMA management [5-7]; furthermore, a preliminary communication is available about another system [8]. The former two are based on off-the-shelf, commercial products, in particular Microsoft Excel and Adobe Photoshop (plus additional software and templates developed by the Authors), and deal with either data and image processing. The third system [7] is based on Web technologies, and it is developed using proprietary, commercial software. The system described in the communication [8] is the closest to our approach, as it is open-source.

There has been also an attempt to standardise the the communication of TMA data, by means of XML (eXtensible Markup Language) [9]; in particular, a working group defined a preliminary DTD (Document Data Type), which can be used to communicate TMA data among different systems. The proposal is very interesting, due to its switch towards openness and communication, and it has been recently adopted by the Cooperative Prostate Cancer Tissue Resource for distributing samples of prostate cancer annotated with demographic and clinical data [10].

Our approach involves the development of a multi-user web-based system, based on open source software, meant at dealing with the management of TMA data from its design to its reporting. The present paper describes the software system we developed and its preliminary user validation.

## Methods

### Design of the system

Following the principles of software engineering, a requirement analysis has been carried out by two Authors, basing on discussions with three pathologists involved in the preparation and analysis of TMA. A basis for the discussion has been the preliminary analysis of the method used at that time, which involved the design of the TMA with the aid of a grid prepared with a word processor, where paraffin block numbers and minimal patient data were put prior to actually prepare the TMA block. The requirement analysis output was a list of specifications, which have then mostly implemented in the system. Part of the specifications resulted then in a entity-relationship scheme and a context diagram, which have been the basis for the underlying database development.

As the TMA is managed by different people (technicians; the pathologist in charge of TMA design; the pathologist in charge of TMA analysis), part of the system is devoted to the management of users.

In order to make the system easily usable in a multi-user context, we decided also to base its interface on web standards. To do this, we had to distinguish a server-side component, which hosts the data, accomplishes to the user authentication, and generates the user interface, and the client side, where the final user accesses the system by means of a web browser.

Among the aims of the study, one was to make the system available on as much as possible operating systems, in order not to lock-in with a single vendor of software. We thus decided to develop it using largely available open source tools, which include:

- PHP as programming language for the logical functions of the system and data access [11];

- MySQL as database management system [12];

- Apache as web server [13];

- any browser as client software.

All the cited software is easily available on the Web (see Table 1), and can run on most current computer systems.

### Preliminary validation

The system has been tested by inputing the data for a project aimed at detection of PTEN and Egr-1. The pathologists participating to the test were briefly trained by the system developer.

Tissue microarrays were performed from identified representative areas of tissues and lesions and, for each case, two 1 mm cores were taken.

## Results

### The developed system

The resulting system, TIMAN (TIssue MicroArray maNagement) is a web-accessible TMA data management system able to cover most of the pathologist' needs. Main features include:

- ability to manage TMAs of different size;

- support to the design and preparation of a TMA: the data related to each paraffin block included in the TMA are inputed before the actual work. The system is then able to provide a print of the TMA scheme, which can be used by the technician in duty of TMA preparation as a map for tissue cores;

- support to immunohistochemistry evaluation: when manually evaluated, percentage of positivity and intensity of staining can be inserted into the database by means of a map where cores already evaluated are marked with a colour different from that of cores still to be evaluated;

- search function on most fields of the database;

- Data export in text format (values separated by tabulator characters, readable by most statistical packages); the set of fields to be exported is configurable;

- a bilingual online help (italian and english), which can be easily extended to other languages:

- a relatively simple installation procedure (although some technical knowledge is needed).

The web interface has been realised by referring to approved Web standards like XHTML 1.0 and CSS2.

The technologies used for the development make TIMAN highly platform independent and interoperable, either on the server as well as on the client side: the server application has been tested with Windows 98, RedHat Linux and MacOSX, while the client interface has been tested with Mozilla 1.2 and Explorer 5–6 on the three main platforms (Windows, Linux, MacOSX). The server software is compatible with PHP 4.0.

**Figure 1:**
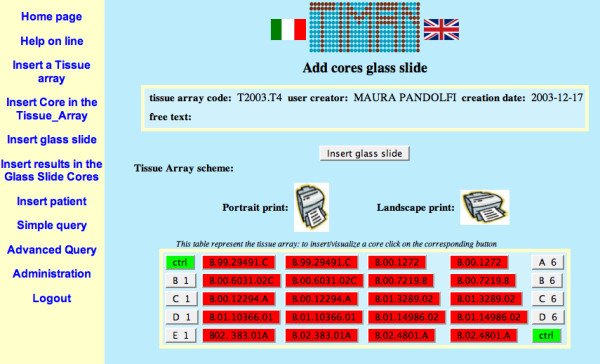
**A screenshot from the software interface**. Page for the input of core data.

The final system is composed by a MySQL database with six tables, and 48 source files (PHP and HTML). Figure 1 shows a screenshot of the system interface.

As privacy of data is an important concern, TIMAN is able to cope at least with some of the related problems by exploiting the HTTPS protocol, which gives to the Web an acceptable level of security at least on the network transmission of data.

### Preliminary validation

During the first steps of data insertion, users asked for slight modifications on the interface and on part of the data model, to meet needs that were not been fully expressed at the design phase, or that appeared after having at disposal an information system instead of the previous paper-based approach. Such modifications included the need for a little controlled terminology where immunohistochemistry antibodies and histochemistry stainings are defined in a uniform way. This to force in the use of unique names for stainings.

Data related to TMAs of 52 paraffin-embedded, formalin-fixed specimens from 6 cases of normal thyroid, 10 cases of nodular hyperplasia, 10 thyroid adenomas, 10 follicular thyroid carcinomas, 10 papillary thyroid carcinomas and 6 undifferentiated thyroid carcinomas were inserted into our system.

At the end of validation, the system hosted six TMA, for a total of 104 cores related to 52 patients. The TMAs were constructed using a tissue arrayer (Beecher Instruments, Silver Spring, MD). TMAs contributed to a study aimed at evaluating PTEN and Egr-1 expression in thyroid proliferative lesions [14].

Pathologists were overall satisfied of the system, mainly because it provides for a number of controls that make easier to prevent errors in data input as well as interpretation.

## Discussion

The proposed system provides for a more focussed number of features in respect to the other freely available systems for TMA [5-8], i.e., it is devoted specifically to the data management. In our opinion, however, TIMAN approaches this side of the problem with greater sensibility, by basing the data storage on a truly relational database management system. On the long term, this allows for greater scalability in respect to a growing number of TMAs. Furthermore, TIMAN takes explicitly into account the multi-user nature of the TMA design and management tasks, from either the data model as well as access capability points of view. The latter is obtained by means of a web interface, which involves the use of a standard web browser to access the system. Although the web interfaces are not always the most efficient ones, it should also be said that the browser is nowadays a universally known tool, which makes training a little less crucial in the use of the system.

A web-based system implies also the use of a web server. This may give some additional technical difficulty in the installation phase, also because the installation procedure that we provide at present is not as simple as such of other software, and in the worst case involves the download and installation of the other software packages needed for TIMAN (i.e., PHP, MySQL, Apache). However, it should be said that Linux and MacosX systems are already provided with the necessary software, while for Windows systems there are a number of packages that install exactly the support software (e.g., PHPtriad), because they represent the typical configuration for a web server.

Regarding the web server, it should be also said that not always the internal information technology procedures allow for the installation of such kind of software, and even if allowed, they might be blocked by personal or local area network firewalls. In these cases an interaction with the responsibles for the network is needed, in order to identify where to place the system (which may co-exist with other software). Finally, there is always the possibility to use the system in a stand-alone configuration, by simply having the server and the client on the same computer.

Anyway, if data that make the patient recognisable are present, then it is better to connect the system to the hospital intranet, rather than on the Internet. On the other side, the proposed system may provide its best if adopted in support to multi-center studies, in which case suitable anonymisation procedures might be needed in order to use the Internet for communication.

The tests carried out by pathologists revealed a number of necessary enhancements, which have been easily implemented in the current release of the software. The process of designing, preparing and analysing a tissue micro array remains an engaging one, due to the large amount of data and the need to correlate them with spatially defined objects like tissue cores. However, TIMAN appears to help in this, at least by avoiding the previously adopted double step of handwriting data and then reporting them on a computer worksheet for processing. Furthermore, an aid comes also by some system interfaces that reproduce the spatial disposition of tissue cores.

We decided not to deal with automatic image processing and data analysis directly into our system; this because we considered that such features are pertaining to very different research fields, and to be eventually implemented with specific software modules (included those developed by us in another project [15]. However, TIMAN can be extended to provide the same features: for example, it could be provided of a web services interface for being automatically accessed by a digital slide acquisition module. Extension is also facilitated by the fact we are distributing the system as open source, under a license that allows for modification and redistribution of the code, provided that the original source is cited. No support is given to installation, due to scarce personnel resources.

Finally, the Tissue Microarray data exchange specification [9] is still not implemented, but it will be one of the future enhancements we will carry out on the system, although we hope that others will try to add this feature by just enhancing the openly released source code.

## Conclusion

A system for tissue microarray data management has been developed, tested on in thyroid proliferative lesions microarrays, and made available to the public in the form of open-source software.

## Availability and requirements

Project name: TIMAN (Tissue MicroArray Management)

Project home page: 

Operating system(s): Platform independent;

Programming language: PHP 4.3; Other requirements: MySQL 3.23 or higher, Apache 1.3 or higher

Licence: GNU GPL.

## Competing interests

The author(s) declare that they have no competing interests.

## Authors' contributions

VDM coordinated the whole project, supervised the developer, and revised the software implementation. IB developed specifications and software as master thesis in Computer Science. MP produced tissue microarrays for the validation and participated in the specification phase as user. CDL was involved as user in the specification phase and coordinated the validation phase.
